# Genomic Insights into Pig Domestication and Adaptation: An Integrated Approach Using Genome-Wide Selection Analysis and Multiple Public Datasets

**DOI:** 10.3390/ani14213159

**Published:** 2024-11-04

**Authors:** Haoyuan Zhang, Pengcheng Ruan, He Cong, Lu Xu, Baigao Yang, Tao Ren, Dongjie Zhang, Hongyue Chen, Pengfei Hu, Zhen Wang, Hongmei Pan, Xiuqin Yang, Yanguo Han, Yan Zeng, Yongju Zhao, Di Liu, Simone Ceccobelli, Guangxin E

**Affiliations:** 1 College of Animal Science and Technology, Southwest University, Chongqing 400715, China; 2 Institute of Animal Husbandry, Heilongjiang Academy of Agricultural Sciences, Harbin 150086, Chinaliudi1963@163.com (D.L.); 3 Chongqing Animal Husbandry Technology Extension Station, Chongqing 401121, China; 4 Institute of Antler Science and Product Technology, Changchun Sci-Tech University, Changchun 130000, China; 5 Chongqing Academy of Animal Sciences, Chongqing 408599, China; 6 College of Animal Science and Technology, Northeast Agricultural University, Harbin 150030, China; 7 Department of Agricultural, Food and Environmental Sciences, Università Politecnica delle Marche, Via Brecce Bianche, 60131 Ancona, Italy

**Keywords:** pig, genome-wide selection, selection signature, major histocompatibility complex, disease-resistant breeding

## Abstract

As a global focus of animal husbandry, pigs provide essential meat resources for humans. Therefore, analyzing the genetic basis of adaptability, domestication, and artificial selection in pigs will contribute to further breeding. This study performed a genome-wide selection sweep analysis to identify candidate genes related to domestication and adaptive selection via data from 2413 public genotypes. Two complementary statistical analyses, *F_ST_* (fixation index) and XP–EHH (cross–population extended haplotype homozygosity) were applied. The results revealed that numerous candidate genes were associated with high-altitude adaptability (e.g., *SIRPA*, *FRS2*, and *GRIN2B*) and habitat temperature adaptability (e.g., *MITF*, *PI3KC2A*, and *FRS2*). In addition, candidate genes related to the domestic genetic imprint of indigenous pigs (e.g., *TNR*, *NOCT*, and *SPATA5*) and strong artificial selection pressure in commercial breeds (e.g., *ITPR2*, *HSD17B12*, and *UGP2*) were identified in this study. Specifically, some MHC–related genes (e.g., *ZRTB12*, *TRIM26*, and *C7H6orf15*) were also under selection during domestication and artificial selection. Additionally, a phylogenetic comparative analysis revealed that the genetic divergence between populations does not fully follow the geographical distribution and management history in the major histocompatibility complex region/major histocompatibility complex II haplotypes, unlike that of the genome-wide genotypes. Furthermore, the higher heterozygosity and haplotype alleles of MHC reduce the differences between populations. Briefly, this study not only helps promote the relative theoretical understanding of environmental adaptive selection and domestication but also provides a theoretical reference for disease-resistant breeding in pigs.

## Simple Summary

Pork is an indispensable meat source for humans. Clarifying the genetic basis of the adaptability and domestication of pigs will promote the breeding process. In this study, numerous candidate genes related to the environmental adaptation, domestication, and artificial selection of pigs were selected via genome-wide selection sweep analysis. In particular, some genes located in major histocompatibility complex regions were also under selection during domestication and artificial selection. Phylogenetic comparative analysis revealed obvious differences in the population distribution and management history between MHC region/MHC II haplotypes and genome-wide genotypes. These findings enhance our theoretical understanding of the environmental adaptability and domestication of pigs and offer valuable insights for disease-resistance breeding in pigs.

## 1. Introduction

As one of the main domestic animals, pigs (*Sus scrofa*) play important roles in agricultural systems worldwide [1]. Zooarchaeological evidence has demonstrated that pigs were domesticated independently in Anatolia and the Yellow River valley of China, dating back at least 8000–10,000 years [2,3]. Since then, various phenotypic differences in the appearance, growth, reproduction, meat quality, and adaptability of pigs have resulted from long-term natural and artificial selection [4]. Currently, pigs provide a stable source of food for humans; moreover, pigs are the main meat consumed worldwide and an important dietary component, especially in Asia [5]. Humans have bred multiple pig breeds that adapt to different feeding environments and meet the expected demands by breeding and improvement [6,7].

Many candidate genes (CDGs) related to pig environmental adaptability and artificial selection for economic breeding have been investigated by sweeping genomic imprinting. For example, *MITF* and *EDNRB* were reported to be associated with the two-end black color trait in Tongcheng pigs [8]. *TLR9* was found to be related to the reproductive traits of Taihu pigs [9]. A genome-wide detection of selection signatures in Duroc pigs revealed that *INSR*, *IGF1R,* and *IGF2R* may be the key CDGs for their excellent growth traits [10]. A series of genes (e.g., *C10orf67*, *VEGFC*, and *ADAMTS9*) enriched in the hypoxia response category were confirmed to be related to the high-altitude adaptation of Tibetan pigs to low-oxygen environments [11,12,13,14]. The *FOXM1* and *RTEL1* genes, which are considerably enriched in the pathway of DNA damage repair, were found to help Tibetan pigs maintain genomic stability during DNA replication and resist UV stimulation [11].

The major histocompatibility complex (MHC), a tightly linked group of genes located in specific regions of vertebrate chromosomes, plays a crucial role in the immune system, and its encoded product is called the major histocompatibility antigen [15]. The main function of the MHC is to recognize invading factors from bacteria, viruses, and other external sources within the body, thereby triggering the immune system to respond accordingly [16,17]. Ample evidence suggests that MHC is not only associated with animal disease resistance and adaptability [18] but also closely linked to the economic traits of livestock [19,20]. In particular, theories regarding the maintenance of genetic diversity and evolutionary drivers of MHC regions in animals remain unclear, which limits the contribution of MHC region variations to disease-resistant breeding in domesticated animals.

However, most previous studies focused on populations with narrow geographical regions or limited management backgrounds. Furthermore, given the presence of multiple copies of genes and gene jumps in MHC regions, current research on MHC polymorphisms is limited to identifying fragment polymorphisms in some key genes [21,22]. Fortunately, commercial high-density genome-wide SNP chips of pigs (Illumina 60k SNP chips) have been widely used worldwide in recent decades [23,24], providing sufficient data for a more comprehensive understanding of the domestication and adaptive selection of domestic pigs. In particular, accurate hybridization signals at each locus in the genome enable the precise localization and acquisition of MHC variant genotypes with large chromosome spans, contributing to the identification of diverse MHC regions. Therefore, this study utilizes publicly available high-density SNP chip data from 2413 pigs across 129 worldwide breeds to identify genomic imprints of domestication and adaptation in domestic pigs, helping breeders further understand the genetic basis of the natural selection and domestication of domestic pigs. In particular, this study emphasizes the role of the major histocompatibility complex (MHC) in these processes, providing breeders further understanding into the genetic mechanisms underlying natural selection and domestication in pigs.

## 2. Materials and Methods

### 2.1. Acquisition of Original Data

A total of 2413 public Illumina 60k SNP datasets (Illumina, San Diego, CA, USA) from 129 pig breeds worldwide were retrieved from two published studies and available in the Dryad Digital Repository [25,26], including 554 wild boars, 1367 indigenous pigs, and 492 commercial individuals. Further details are provided in Table S1. The original datasets were combined via the PLINK v1.90—*merge* command.

### 2.2. Genome-Wide Selection Signal Analysis

The original datasets were filtered by VCFtools (v0.1.16) with a minor allele frequency (MAF) of <0.05 and a call rate of ≥0.9, and nonbiallelic SNPs were removed. A total of 44,081 autosomal SNPs were subsequently retained for analysis. Multiple pig breeds were used for genome-wide selection signal analysis (GWSA) to screen candidate genes associated with high altitude adaptability (case: altitude > 2800 m, control: altitude < 300 m), habitat temperature adaptability (case: South China breeds, control: North China breeds), domestication from wild boars to indigenous pigs (case: wild boars, control: indigenous pigs), and artificial selection of indigenous pigs to commercial breeds (case: indigenous pigs, control: commercial breeds) (Table S2). With respect to GWSA, the Pairwise Fixation Index (*F*_ST_) [27] and cross-population-extended haplotype homozygosity test (XP-EHH) [28] were performed via VCFtools (v0.1.16) and selscan (v2.0.0, https://github.com/szpiech/selscan (accessed on 10 October 2023)), respectively. CDGs were defined as the intersection of genes annotated by SNPs with a top 1% threshold of *F*_ST_ and a top and tail 1% threshold of XP-EHH based on the reference genome annotation file (*Sus scrofa* 10.2, GCA_000003025.4). Kyoto Encyclopedia of Genes and Genomes (KEGG) and Gene Ontology (GO) analyses of the CDGs were performed using KOBAS 3.0 (http://bioinfo.org/kobas (accessed on 15 June 2024)).

### 2.3. Genetic Diversity Characteristics of Pig MHC and Genome-Wide Data

The original datasets were filtered with parameters of MAF < 0.05, a call rate of ≥0.95, and the PLINK function *indep-pairwise* 50 10 0.1 by PLINK software, resulting in 6929 SNPs for subsequent population phylogenetic analysis and estimation of the diversity of all the animals at the genome-wide level. In contrast, 63 SNPs were retained from the pig MHC I (27 SNPs), II (16 SNPs), and III (19 SNPs) molecular regions.

The *p*-distance matrix between individuals was estimated by VCF2Dis (https://github.com/hewm2008/VCF2Dis (accessed on 20 August 2024)). The neighbor-joining phylogenetic tree was constructed with FastME v2.1.6.2 software [29] and visualized with iTOL (https://itol.embl.de/ (accessed on 20 August 2024)). Principal component analysis (PCA) was performed via PLINK and visualized with the R program (ggplot2 package). The linkage disequilibrium (LD) of the genomic region was determined via HaploView 4.2 (https://www.broadinstitute.org/haploview/haploview (accessed on 20 July 2024)). Population phylogenetic clustering was performed using ADMIXTURE v1.3.0 (https://dalexander.github.io/admixture/ (accessed on 20 July 2024)) with *K* = 2–10, and the minimum CV error of the *K* value was considered the most reliable *K* value. The haplotypes of MHC II were constructed by DnaSP v6 (http://www.ub.edu/dnasp/ (accessed on 15 October 2023)) using the phase method. The observed heterozygosity (*H*_O_), expected heterozygosity (*H*_E_) of the population, and pairwise differences (*F*_ST_) were estimated using Arlequin v3.5.2.2 software (https://cmpg.unibe.ch/software/arlequin35/ (accessed on 20 July 2024)) with default parameters. Correlation coefficient analysis between these parameters was performed via R package ggplot2.

## 3. Results

### 3.1. Genome-Wide Selective Sweep Analysis of Environmental Adaptability and Domestication in Pigs

**Figure 1:**
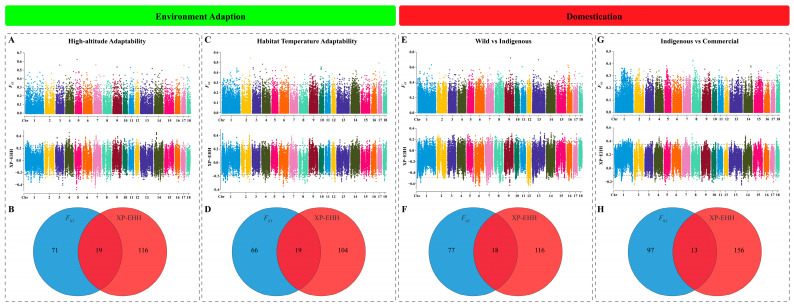
Manhattan plots of selection signatures for environment adaptability and domestication of pigs using *F*_ST_ and XP–EHH methods. (**A**) Manhattan plots for high-altitude adaptability. (**B**) A Venn diagram of the intersection of candidate genes for high-altitude adaptability. (**C**) Manhattan plots for habitat temperature adaptability. (**D**) A Venn diagram of the intersection of candidate genes for habitat temperature adaptability. (**E**) Manhattan plots for domestication from wild boars to indigenous pigs. (**F**) A Venn diagram of the intersection of candidate genes for domestication from wild boars to indigenous pigs. (**G**) Manhattan plots for artificial selection from indigenous pigs to commercial pigs. (**H**) A Venn diagram of the intersection of candidate genes for artificial selection from indigenous pigs to commercial pigs.

First, a total of 19 CDGs (e.g., *CYP46A1*, *GRIN2B*, and *PDE9A*) associated with adaptation to high-altitude environments in pigs were identified (Figure 1A). A total of 26 KEGG pathways and 238 GO terms were enriched. Notably, some CDGs (e.g., *FRS2*, *PDE9A*, and *SIRPA*) were enriched in the KEGG pathways associated with the nervous system and in the GO terms related to vascular and cardiac muscle (Table S3). Second, a total of 19 CDGs (e.g., *ASRGL1*, *PIK3C2A*, and *MITF*) related to pig environmental temperature adaptability were obtained (Figure 1C). These genes were enriched in 37 KEGG pathways and 199 GO terms, especially melanin- and metabolism-related pathways, and GO terms related to the nervous system (Table S4). With respect to the domestication of pigs, 18 CDGs (e.g., *THEMIS*, *PRIM2*, and *NR5A2*) were identified as being involved in the domestication of wild boars into indigenous pig breeds (Figure 1E). These genes were enriched in 14 KEGG pathways and 191 GO terms. According to the enrichment results (Table S5), several pathways and GO terms, such as brain development, circadian rhythms, and spermatogenesis, attracted our attention. Subsequently, 13 CDGs (e.g., *SND1*, *ITPR2*, and *HSD17B12*) were associated with the artificial selection breeding of indigenous pigs into commercial breeds (Figure 1G). A total of 53 KEGG pathways and 127 GO terms were enriched by these CDGs (Table S6). Interestingly, *HSD17B12*, *UGP2,* and *ITPR2* were enriched in the KEGG pathways related to the nervous system, the synthesis and secretion of sex hormones, and the metabolism of carbohydrates.

### 3.2. Genetic Diversity Characteristics of Pig MHC at Different Domestication and Selection Stages

**Figure 2:**
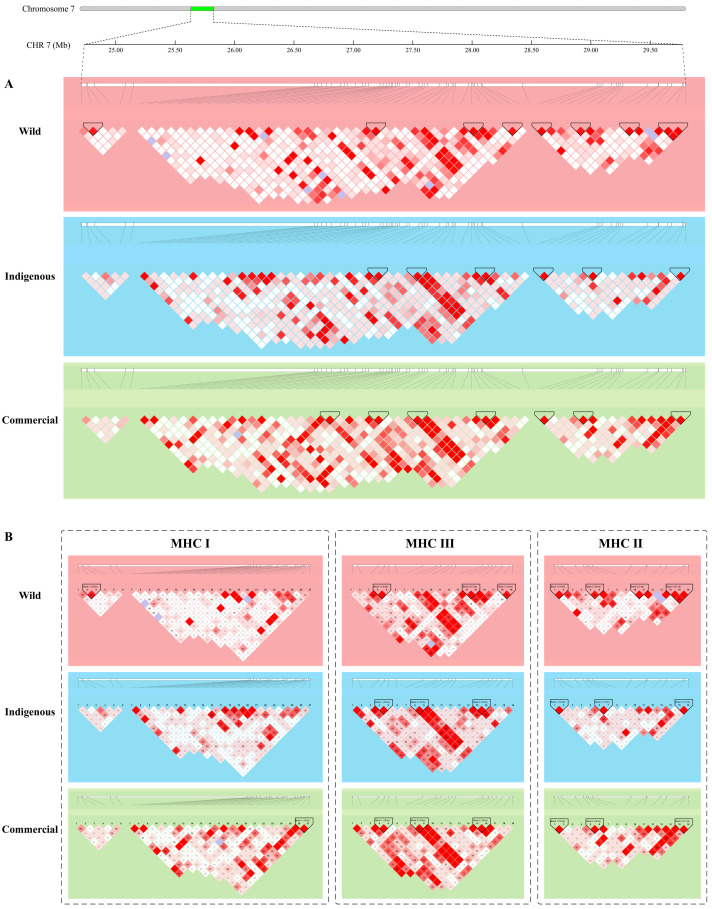
Linkage disequilibrium in the MHC region of pigs. (**A**) Pairwise linkage disequilibrium plots of MHC in pigs. (**B**) Pairwise linkage disequilibrium plots of MHC I, MHC II, and MHC III in pigs.

An LD block analysis of the MHC regions revealed a block between CHR7_27,778,363 bp and CHR7_27,797,198 bp in the MHC III region in indigenous and commercial pigs, which cover the *C2* and *ZRTB12* genes (Figure 2B). A unique block between CHR7_27,373,527 bp and CHR7_27,389,510 bp from the MHC I region, which included the *C7H6orf15*, *DPCR1,* and *C7H6orf205* genes, was investigated in commercial breeds. Additionally, two blocks were confirmed only within wild boars: one is CHR7_24,777,963 bp and CHR7_24,791,155 bp in MHC I (*LOC100154127* and *TRIM26*), and the other is CHR7_29,543,539 bp and CHR7_29,546,337 bp in MHC II (*BRD2* and *SLA-DQA*).

**Figure 3:**
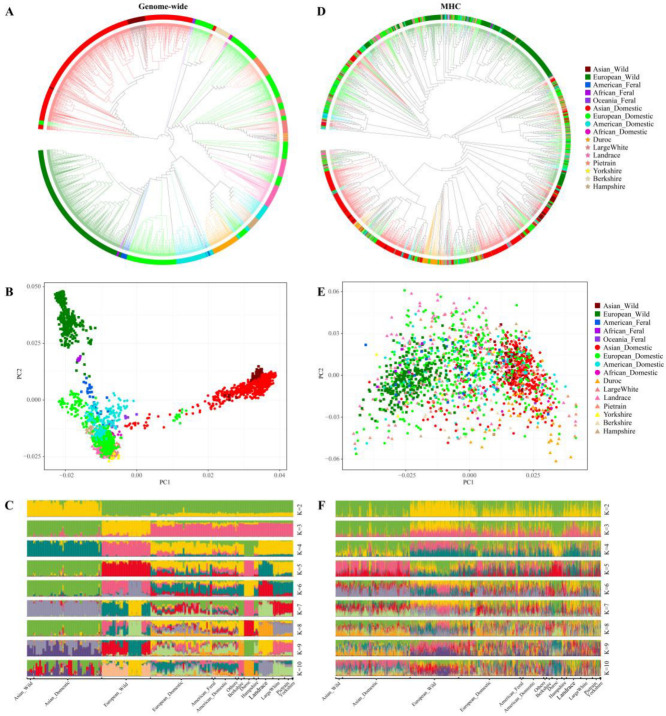
Genetic structure of worldwide pigs. (**A**) Neighbor-joining tree of pigs constructed by genome-wide data. (**B**) Plots of principal components analysis based on genome-wide data. (**C**) Admixture results of worldwide pig breeds from *K* = 2–10 based on genome-wide data. (**D**) Neighbor-joining tree of pigs constructed by MHC data. (**E**) Plots of principal components analysis based on MHC data. (**F**) Admixture results of worldwide pig breeds from *K* = 2–10 based on MHC data.

Furthermore, the phylogenetic tree of the genome-wide genotypes revealed that nearly all individuals from each population or breed were classified together, indicating that their phylogenetic relationships are consistent with their geographical distribution and management history (Figure 3A). Specifically, the commercial breeds bred separately in Europe (e.g., Landrace, Pietrain, and Yorkshire) and America (Hampshire and Duroc) also presented distinct geographical origins. Similarly, the same population structure and individual kinship patterns were presented by both the PCA and ADMIXTURE results from the genome-wide data (Figure 3B,C). In particular, the differences between groups became more pronounced as the *K* value increased (Figure 3C). In contrast, the phylogenetic tree constructed from the MHC data revealed a mixture of populations from different geographical distributions (Figure 3D). The PCA results also revealed that pigs from different continents presented greater genetic similarity compared to the genome-wide data (Figure 3E). The ADMIXTURE results revealed that the most reliable *K* value of the MHC region was 7, and the genetic stratification of all populations gradually became more complex as the *K* value increased (Figure 3F).

**Figure 4:**
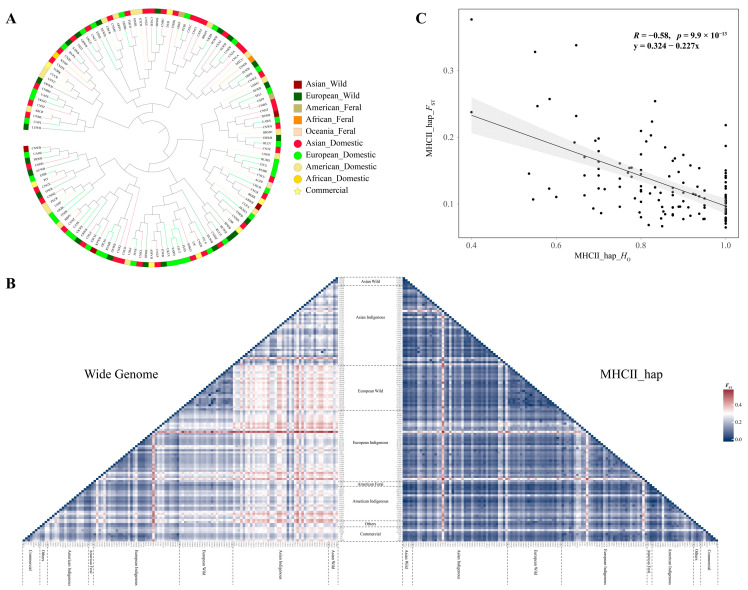
Phylogenetic network and population differentiation of 129 pig populations based on genome-wide data and MHC II haplotypes. (**A**) Neighbor-joining tree of 129 pig populations constructed by the haplotypes of MHC II. (**B**) Pairwise population differentiation between 129 pig populations based on the genome-wide data and the haplotypes of MHC II. (**C**) Correlation between observed heterozygosity and mean *F*_ST_ of MHC II haplotypes in 129 pig populations.

Additionally, a total of 441 haplotypes were reconstructed by 16 SNPs from the MHC II region. The population phylogenetic tree revealed more chaotic population relationships than individual-level relationships with MHC SNPs (Figure 4A). Smaller *F*_ST_ pairs between populations were observed from haplotypes of MHC II than those of the genome-wide genotypes (Figure 4B). Furthermore, the MHC II haplotype *H*_O_ of all populations was uniformly greater than that of the genome-wide genotypes (Table S7), and the mean *F*_ST_ of the MHC II haplotype in more than 99% of the populations was lower than that of the genome-wide genotypes (Table S8). Finally, the correlation analysis results between *H*_O_ and the mean *F*_ST_ of the MHC II haplotype for each population revealed a significant negative correlation (Figure 4C, *R* = −0.58, *p* = 9.9 × 10^−13^).

## 4. Discussion

### 4.1. Genetic Basis of High-Altitude Adaptability in Eurasian Pigs

As one of the important livestock species in the Qinghai–Tibet Plateau, Tibetan pigs play an important role in the regional agricultural economy. Importantly, thousands of years of natural selection have enhanced their ability to adapt to the challenges of high-altitude environments, including hypoxia and intense ultraviolet radiation [13]. Therefore, Tibetan pigs are suitable animal models for understanding the genetic mechanism of high-altitude adaptation. A series of biological characteristics formed under long-term natural selection, such as good cardiopulmonary development, loss of hypoxic pulmonary vasoconstriction, and right ventricular hypertrophy, contribute to the survival of plateau animals [30,31]. In this study, a series of CDGs were enriched in the GO terms related to cardiomyocytes and angiogenesis. Nitric oxide plays a major role in the changes in vascular tone and organ function under hypoxia [32], whereas *SIRPA* is characterized as an acute regulator of arterial vasodilation, which can inhibit nitric oxide-mediated vasodilation [33]. *FRS2* has been found to regulate the differentiation of human pluripotent cardiovascular progenitors and cardiomyocytes [34], while the inhibition of *PDE9A* reduces cardiac hypertrophy through natriuretic peptide-dependent cGMP-PKG signal transduction [35], all of which may be related to the increased heart size of Tibetan pigs. *THSD7A*, which is involved in embryonic angiogenesis [36], has also been reported to be related to the adaptation of Tibetan pigs to the hypoxic environment in previous studies [37]. The enrichment of the *GRIN2B* gene in multiple pathways of the nervous system category also caught our attention. *GRIN2B* has been reported to be associated with high-altitude adaptation in Tibetan pigs [31]. The *FRS2* gene was also enriched in the thermogenesis pathway, which may help Tibetan pigs maintain body temperature at high altitudes, but no relevant findings have been reported in previous studies.

### 4.2. Genetic Basis of Habitat Temperature Adaptability in Eurasian Pigs

In recent years, the increasing frequency of extreme weather events worldwide underscores the urgent need to investigate the genetic basis of temperature adaptation in pigs. The vast geographical expanse of China allows indigenous pig breeds, which have thrived at various latitudes and altitudes, to exhibit distinct environmental adaptability. This study aimed to explore the genetic basis of temperature adaptation in pigs by comparing the genomic differences between native pig breeds from Southern China and those from Northern China. In this study, numerous CDGs were observed to explain the adaptability of pigs to habitat temperature. *MITF* is a key gene in the melanogenesis pathway [38]. Heat stress can markedly increase the expression of *MITF*, resulting in increased melanogenesis [39]. The coat color plays an important role in protecting animals from environmental stresses, such as high temperatures. The profound effects of environmental temperature on biological processes, such as aerobic metabolism, have been demonstrated in a previous study [40]. Cellular metabolism is the basis of all biological activities [41]. Studies have shown that the *PI3KC2A* [42], *ASRGL1* [43], and *CHSY1* [44] genes are involved mainly in metabolism by regulating cellular proliferation. The *PIK3C2A* and *FLI1* genes were enriched in the GO terms related to blood circulation and angiogenesis. Notably, thermal adaptation is achieved by increasing blood flow to the skin, which increases heat loss [45]. Several CDGs (e.g., *FRS2*, *TRIM2*, and *GRIN2B*) were enriched in multiple GO terms related to forebrain development, neurons, and synapses, which is consistent with the fact that temperature changes have considerable effects on the function of the nervous system and its components [46]. Interestingly, two CDGs (*FRS2* and *GRIN2B*) associated with both the high-altitude adaptability and the habitat temperature adaptability of pigs were identified. The climates in high-altitude areas and high-latitude areas of Northern China are relatively cold. Therefore, these common CDGs may be related to the thermoregulation of indigenous breeds in low-temperature environments.

### 4.3. Domestic Genetic Imprinting of Indigenous Pigs from Wild Boars

Since wild boars were domesticated in Eurasia, various phenotypic differences in the appearance, growth, reproduction, and meat quality of pigs have resulted from long-term natural and artificial selection [4]. In this study, wild boar and indigenous breeds from all over the world were utilized to identify candidate genes associated with the domestication of pigs. The results revealed that some CDGs (*TNR*, *NOCT*, and *SPATA5*) are involved in the GO terms of synaptic transmission, circadian rhythms, and brain development. The changes in brain size occurred during domestication, which may be related to the relaxed selection pressure on cognitive abilities in the human environment [47]. In addition, various biological functions, such as metabolism and cell proliferation, are regulated by circadian rhythms [48,49]. Evidence shows that domestic pigs can adapt to photoperiod changes by changing their circadian rhythm through the secretion of melatonin [50]. In particular, *NOCT* controls specific circadian pathways associated with the uptake and utilization of lipids [51]. In addition, *RLF* and *SPATA5* play important roles in spermatogenesis and testis development [52,53], which is in agreement with the knowledge of differences in reproductive traits between wild boar and domestic pigs during domestication [54].

### 4.4. Genetic Imprinting of Strong Artificial Selection Pressure in Commercial Breeds Worldwide

Over the past 200 years, a series of high-economic-value traits have been rapidly fixed and standardized in commercial pig breeds through intense artificial selection. These commercial breeds, such as Large White, Landrace, and Duroc breeds, present advantages, including fast growth rates, superior meat quality, and high feed conversion efficiency [55]. This study aims to perform whole-genome selection signal scanning on these breeds to reveal the genomic imprints resulting from strong artificial selection. In this study, numerous CDGs related to the strong artificial selection of commercial pig breeds were identified, with the aim of explaining the genetic roots in the context of commercial breeding. *ITPR2* was enriched in multiple pathways of the nervous system category. The knockout of *ITPR2* led to abnormalities in the striatum, a key component of the emotion-regulating network in mice, which may be related to the depression-like behavior associated with *ITPR2* deficiency [56]. Moreover, several CDGs (*ITPR2*, *HSD17B12*, and *UGP2*) enriched in KEGG pathways are associated with the synthesis and secretion of sex hormones. A previous study suggested that an insufficient dose of *HSD17B12* affects the fertility of female mice, resulting in premature ovarian insufficiency [57]. *UGP2* was reported to be related to the semen quality of Niangya yaks [58]. In addition, several CDGs were enriched in KEGG pathways, such as salivary secretion, gastric acid secretion, and metabolism of multiple carbohydrates, which may be related to the digestive function and absorption capacity of commercial pig breeds.

### 4.5. Genetic Diversity Characteristics of Pig MHC at Different Domestication and Selection Stages

The MHC is a term referring to a group of genes encoding major histocompatibility antigens in animals whose main function is antigen presentation [59]. An increasing number of studies have confirmed that MHC is related not only to immunity but also to the multiplex traits of domesticated animals [60,61]. In this study, an LD block covering three genes (*STG*, *MUCL3*, and *C7H6orf205*) was observed in commercial pigs compared with indigenous/wild pigs. In clinical practice, *MUCL3* is considered a prognostic marker for immunogenic cell death associated with patients with colorectal cancer [62]. Specifically, *MUCL3* has been confirmed to be associated with cancers and neuropsychiatric disorders [63,64]. Commercial pigs suffer from negative experiences of life emotions and the expression of animal instincts due to industrial management [65].

In addition, an LD block covering two genes (*C2* and *ZBTB12*) was shown in indigenous/commercial animals. Recently, complementary *C2* may be causally related to increased type 2 diabetes risk [66,67]. A previous study confirmed that the upregulation of *C2* is positively correlated with obesity and hyperinsulinemia in subcutaneous adipose tissue and adipocytes and negatively correlated with the expression of insulin signaling-related genes [68]. In addition, *ZBTB12* plays important roles in various biological functions, such as serving as a molecular barrier that protects the unidirectional transition from the fate of metastable stem cells to the late developmental stage [69], and *ZBTB12* methylation is associated with cardiovascular disease risk [70]. In particular, a large haploblock (covering *ZBTB12*) from the human MHC region HLA-B*57 was verified to be related to the HIV viral load [71]. This result suggests that the strong selection under both gene regions in indigenous pigs may help contribute to their different environmental adaptabilities and body shape formations compared with those of wild boars.

The results of both the phylogenetic analysis and the population structure analysis (*K* = 2) based on the genome-wide data indicated that the phylogenetic relationships among 129 worldwide pig breeds aligned with their geographical distribution. Notably, two distinct branches representing European and Asian ancestry were identified, which corroborates previous findings that the domestication of pigs primarily originated in Europe and Asia [25,72]. Furthermore, the differences in phylogenetic analysis results between genome-wide data and the MHC region confirmed the previous discovery of divergence in the evolutionary stage between the MHC region and the autosomal genome [73,74]. In theory, abundant evidence suggests that the MHC region of vertebrates is influenced by multiple selection and evolutionary driving forces [75,76]. This study revealed that the potential dominant genotypes of these MHC-related genes were gradually fixed in the domestication and artificial selection stages. Some evidence supports the argument that the MHC suffers from positive selection [77,78]. However, domestication does not significantly reduce MHC diversity in pigs [79]. Additional evidence suggests that the diversity of MHCs is modified by a combination of complex evolutionary forces, such as genetic drift, balanced selection, pathogen drive, the environment, and migration [73,80,81].

In this study, although the phylogenetic tree and PCA results of the MHC regions did not show a clear phylogenetic relationship consistent with the geographic distribution and management history of the genome-wide genotypes, two clear clusters in Europe and Asia were still observed. This finding supports the hypothesis that multiple forces are involved in maintaining MHC diversity, particularly with a wealth of evidence indicating that the epidemiological history of different geographical regions is crucial to the development of MHC diversity in populations [82]. Additional evidence also suggests that MHC diversity is influenced by ancestral haplotypes, leading to different geographic distribution groups carrying private MHC genotypes inherited from their ancestral animals in the early stages of domestication or their ancient/wide ancestors [83,84] because of the conserved immunological function of the MHC.

Additionally, this study revealed that numerous indigenous and commercial individuals from different geographical distributions exhibit inexplicable close blood relationships in the MHC. A lower population genetic divergence and greater heterozygosity of the MHC region than the genome-wide genotypes were observed, which directly demonstrates that balanced selection is a key force in maintaining the genetic diversity of the MHC. Studies have confirmed that MHC gene loci carry as many alleles as possible through balanced selection, enabling them to bind to multiple pathogen antigens, thereby enhancing the host immune response to pathogens [85,86]. In particular, European rabbit individuals with MHC DRB heterozygous genotypes have greater body weights and lower hepatic *coccidia* loads than homozygous individuals [87]. Therefore, high heterozygosis and more genotypes in the MHC may contribute to different individuals who may share the same alleles and reduce genetic differences between populations/breeds [74]. This is also determined by the unique biological function of the MHC, which plays a critical role in ensuring the survival of animals in natural environments and supporting population persistence.

## 5. Conclusions

This study provides comprehensive insights into the domestication and environmental adaptation of pigs through a genome-wide selection analysis of 2413 pigs across 129 global populations. Numerous CDGs associated with high-altitude adaptation, temperature adaptability, and domestication processes have been identified, shedding light on the genetic mechanisms behind these traits. These findings highlight the complexity of pig domestication, which is driven by both natural and artificial selection pressures. Moreover, this study underscores the importance of the major histocompatibility complex in maintaining genetic diversity across different stages of domestication, with balanced selection playing a crucial role in preserving MHC polymorphisms. These results not only provide a theoretical understanding of pig genetics but also offer valuable information for breeding programs aimed at improving disease resistance and adaptability in domestic pig populations.

## Data Availability

The original data presented in the study were obtained in the Dryad Digital Repository from two published studies (https://datadryad.org/stash/dataset/doi:10.5061/dryad.30tk6 and https://datadryad.org/stash/dataset/doi:10.5061/dryad.8bf48 (accessed on 10 June 2022)).

## Supplementary Materials

The following supporting information can be downloaded at https://www.mdpi.com/article/10.3390/ani14213159/s1, Table S1: Detailed information of pig populations; Table S2: Sample grouping information of genome-wide selection signal analysis; Table S3: Enrichment results of KEGG pathways and GO terms for candidate genes associated with high-altitude adaptability; Table S4: Enrichment results of KEGG pathways and GO terms for candidate genes associated with habitat temperature adaptability; Table S5: Enrichment results of KEGG pathways and GO terms for candidate genes associated with domestication from wild boars to indigenous pigs; Table S6: Enrichment results of KEGG pathways and GO terms for candidate genes associated with artificial selection from indigenous pigs to commercial pigs; Table S7: Comparison of observed heterozygosity between genome-wide genotypes and MHC II haplotypes in all populations; Table S8: Comparison of mean FST between genome-wide genotypes and MHC II haplotypes in all populations.
